# 1-(5-Hydroxy-1-phenyl-3-trifluoromethyl-1*H*-pyrazol-1-yl)ethanone

**DOI:** 10.1107/S1600536809051745

**Published:** 2009-12-09

**Authors:** Hugo Gallardo, Edivandro Girotto, Adailton J. Bortoluzzi

**Affiliations:** a Depto. de Química - Universidade Federal de Santa Catarina, 88040-900 - Florianópolis, SC, Brazil

## Abstract

The crystal structure of the title compound, C_12_H_9_F_3_N_2_O_2_, contains two independent mol­ecules in the asymmetric unit. The mol­ecules are chemically identical but exhibit a significant difference in the dihedral angles between the mean planes of the phenyl and pyrazole rings, with values of of 11.62 (13) and 18.17 (11)°. Moreover, the trifluoro­methyl group in one of the mol­ecules shows rotational disorder of the F atoms, with site occupancy factors of 0.929 (6) and 0.071 (6). The hydroxyl group in each of the mol­ecules shows a strong intra­molecular hydrogen bond with the carbonyl O atom, forming a six-membered ring and forcing the formyl group and pyrazole ring to be coplanarshowing C—C—C—O torsion angles of ?0.3(5)o and 0.°. Weak inter­molecular C—H⋯O and C—H⋯F inter­actions contribute to the stabilization of the crystal packing.

## Related literature

For the pharmaceutical activity of pyrazole derivatives, see: Belmar *et al.* (2001 ▶). For related structures, see: Gallardo *et al.* (2009 ▶); Belmar *et al.* (2006*a* ▶,*b* ▶); Pérez *et al.* (2005 ▶). For the melting point, see: Bieringer & Holzer (2006 ▶).
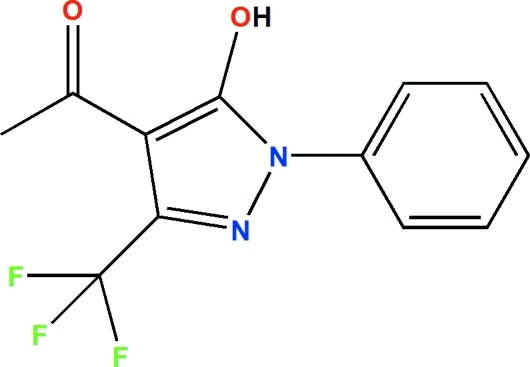


## Experimental

### 

#### Crystal data

C_12_H_9_F_3_N_2_O_2_
*M* *_r_* = 270.21
Triclinic, 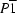

*a* = 7.4779 (19) Å
*b* = 11.9390 (18) Å
*c* = 13.8587 (14) Å
α = 78.591 (11)°
β = 80.090 (17)°
γ = 78.791 (18)°
*V* = 1178.2 (4) Å^3^
*Z* = 4
Mo *K*α radiation
μ = 0.14 mm^−1^
*T* = 293 K
0.40 × 0.33 × 0.13 mm

#### Data collection

Enraf–Nonius CAD-4 diffractometer
4766 measured reflections
4567 independent reflections
2268 reflections with *I* > 2σ(*I*)
*R*_int_ = 0.025
3 standard reflections every 200 reflections
intensity decay: 1%

#### Refinement

*R*[*F* ^2^ > 2σ(*F* ^2^)] = 0.055
*wR*(*F* ^2^) = 0.165
*S* = 1.04
4567 reflections
373 parameters
81 restraints
H-atom parameters constrained
Δρ_max_ = 0.20 e Å^−3^
Δρ_min_ = −0.21 e Å^−3^

### 

Data collection: *CAD-4 Software* (Enraf–Nonius, 1989 ▶); cell refinement: *CAD-4 Software*; data reduction: *HELENA* (Spek, 1996 ▶); program(s) used to solve structure: *SIR97* (Altomare *et al.*, 1999 ▶); program(s) used to refine structure: *SHELXL97* (Sheldrick, 2008 ▶); molecular graphics: *PLATON* (Spek, 2009 ▶); software used to prepare material for publication: *SHELXL97*.

## Supplementary Material

Crystal structure: contains datablocks global, I. DOI: 10.1107/S1600536809051745/pv2233sup1.cif

Structure factors: contains datablocks I. DOI: 10.1107/S1600536809051745/pv2233Isup2.hkl

Additional supplementary materials:  crystallographic information; 3D view; checkCIF report

## Figures and Tables

**Table 1:** Hydrogen-bond geometry (Å, °)

*D*—H⋯*A*	*D*—H	H⋯*A*	*D*⋯*A*	*D*—H⋯*A*
O5—H5⋯O13	1.18	1.60	2.548 (3)	132
O5′—H5′⋯O13′	1.07	1.65	2.560 (4)	139
C14—H14*B*⋯F1′^i^	0.96	2.55	3.394 (5)	146
C7—H7⋯O13^ii^	0.93	2.64	3.446 (4)	145
C7′—H7′⋯O13′^iii^	0.93	2.57	3.406 (4)	149

Symmetry codes: (i) 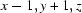; (ii) 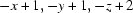; (iii) 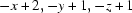.

**Fig. 1.**
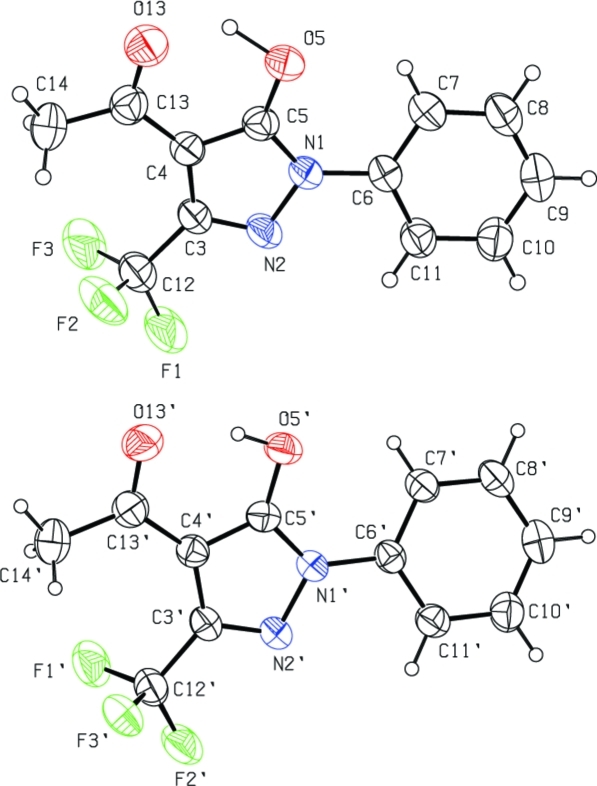
The structure of the two molecules of the title compound with labeling scheme; displacement ellipsoids are shown at the 40% probability level and atoms F1", F2" and F3" representing the smaller fraction of the disordered trifluoromethyl group have been excluded.

## supplementary crystallographic information

### Comment

The pyrazolones are derivatives of pyrazole having an additional carbonyl/hydroxy group. Some derivatives of pyrazolone show analgesic and anti-inflamatory properties (Belmar *et al.*, 2001). In this article, the crystal structure of the title compound (I), which is a derivative of pyrazole is presented.

The asymmetric unit of the title compound contains two independent molecules (Fig. 1). The molecules are chemically identical, but show different spacial conformation. This can be evidenced by the different dihedral angle between the mean plane of the phenyl and pyrazole rings of 11.62 (13)° and 18.17 (11)°. In the pyrazole rings of the two molecules the single bond distances are shorter and the double bond distances are longer that expected values indicating that the pyrazole rings are delocalized π-systems. A cyclic six-membered intramolecular hydrogen bond between hydroxyl and acyl groups force the acyl groups and the pyrazole rings to be coplanar, in both molecules. Several weak intermolecular C—H···O and C—H···F hydrogen bonds are involved in the stabilization of the crystal packing. The crystal structures of a few compounds closely related to (I) have been reported (Gallardo *et al.*, 2009; Belmar *et al.*, 2006*a,b*); Pérez *et al.*, 2005).

### Experimental

In a three necked flask of 50 ml equipped with condenser, were transferred 2-phenyl-5-(trifluoromethyl)pyrazol-3(2*H*)-one (1.5 g, 6.57 mmol), 1,4-dioxane (dried) (25 ml) and Ca(OH)_2_ (0.67 g, 13.1 mmol). The reaction mixture was heated to 373 K with stirring for 15 min, then cooled to room temperature and added slowly ac. acetyl chloride (0.56 g, 7.2 mmol) and allowed to reflux for 24 h. After the reaction was completed, the product was poured into 40 ml of HCl solution (3 mol l^-1^) and ice and stirred for 30 min, resulting in precipitate which was filtered. The compound was purified through a silica gel chromatographic column (eluent: hexane/AcOEt 3%) which yielded a yellow solid (0.525 g) of the title compound. In the purification process, 0.752 g of 2-phenyl-5-(trifluoromethyl)pyrazol-3(2*H*)-one was also recovered.

### Refinement

H atoms were placed at their idealized positions with distances of 0.93 and 0.96 Å and *U*_eq_ fixed at 1.2 and 1.5 times *U*_iso_ of the preceding atom for C—H_Ar_ and CH_3_, respectively. The H atoms of the hydroxyl groups were found from Fourier difference map and fixed at those positions with *U*_eq_ fixed at 1.2 times *U*_iso_ of the parent atom. Trifluoromethyl group of one molecule exhibited rotational disorder with two alternative positions for F atoms. The refined site occupancies for disordered atoms are 0.929 (6) and 0.071 (6).

### Crystal data

**Table:**

C_12_H_9_F_3_N_2_O_2_	*Z* = 4
*M**_r_* = 270.21	*F*(000) = 552
Triclinic, *P*1	*D*_x_ = 1.523 Mg m^−^^3^
Hall symbol: -P 1	Mo *K*α radiation, λ = 0.71069 Å
*a* = 7.4779 (19) Å	Cell parameters from 25 reflections
*b* = 11.9390 (18) Å	θ = 5.4–18.6°
*c* = 13.8587 (14) Å	µ = 0.14 mm^−^^1^
α = 78.591 (11)°	*T* = 293 K
β = 80.090 (17)°	Prismatic, yellow
γ = 78.791 (18)°	0.40 × 0.33 × 0.13 mm
*V* = 1178.2 (4) Å^3^	

### Data collection

**Table:**

Enraf–Nonius CAD-4 diffractometer	*R*_int_ = 0.025
Radiation source: fine-focus sealed tube	θ_max_ = 26.0°, θ_min_ = 1.5°
graphite	*h* = −9→9
ω/2θ scans	*k* = −14→14
4766 measured reflections	*l* = −17→0
4567 independent reflections	3 standard reflections every 200 reflections
2268 reflections with *I* > 2σ(*I*)	intensity decay: 1%

### Refinement

**Table:**

Refinement on *F*^2^	Primary atom site location: structure-invariant direct methods
Least-squares matrix: full	Secondary atom site location: difference Fourier map
*R*[*F*^2^ > 2σ(*F*^2^)] = 0.055	Hydrogen site location: inferred from neighbouring sites
*wR*(*F*^2^) = 0.165	H-atom parameters constrained
*S* = 1.04	*w* = 1/[σ^2^(*F*_o_^2^) + (0.0571*P*)^2^ + 0.4943*P*] where *P* = (*F*_o_^2^ + 2*F*_c_^2^)/3
4567 reflections	(Δ/σ)_max_ = 0.001
373 parameters	Δρ_max_ = 0.20 e Å^−^^3^
81 restraints	Δρ_min_ = −0.20 e Å^−^^3^

### Fractional atomic coordinates and isotropic or equivalent isotropic displacement parameters (Å^2^)

**Table:**

	*x*	*y*	*z*	*U*_iso_*/*U*_eq_	Occ. (<1)
N1	0.4429 (3)	0.5800 (2)	0.73982 (18)	0.0517 (7)	
N2	0.4653 (4)	0.6690 (2)	0.66084 (18)	0.0585 (7)	
C3	0.4755 (5)	0.7585 (3)	0.7002 (2)	0.0557 (8)	
C4	0.4619 (4)	0.7333 (3)	0.8049 (2)	0.0524 (8)	
C5	0.4399 (4)	0.6175 (3)	0.8262 (2)	0.0536 (8)	
O5	0.4173 (3)	0.55236 (19)	0.91394 (16)	0.0695 (7)	
H5	0.4804	0.6027	0.9610	0.083*	
C6	0.4214 (4)	0.4699 (3)	0.7213 (2)	0.0508 (8)	
C7	0.4301 (5)	0.3738 (3)	0.7950 (3)	0.0668 (10)	
H7	0.4494	0.3792	0.8583	0.080*	
C8	0.4099 (5)	0.2695 (3)	0.7735 (3)	0.0749 (11)	
H8	0.4140	0.2046	0.8233	0.090*	
C9	0.3840 (5)	0.2594 (3)	0.6807 (3)	0.0738 (11)	
H9	0.3725	0.1881	0.6670	0.089*	
C10	0.3752 (5)	0.3559 (3)	0.6079 (3)	0.0718 (10)	
H10	0.3567	0.3499	0.5446	0.086*	
C11	0.3934 (5)	0.4620 (3)	0.6275 (2)	0.0633 (9)	
H11	0.3869	0.5271	0.5779	0.076*	
C12	0.4960 (7)	0.8687 (3)	0.6304 (3)	0.0751 (11)	
F1	0.5010 (8)	0.8597 (3)	0.5363 (2)	0.1229 (17)	0.929 (6)
F1''	0.362 (5)	0.899 (4)	0.585 (3)	0.119 (13)	0.071 (6)
F2	0.3541 (5)	0.9518 (2)	0.6488 (3)	0.1112 (14)	0.929 (6)
F2''	0.508 (7)	0.955 (3)	0.667 (3)	0.106 (11)	0.071 (6)
F3	0.6442 (6)	0.9097 (3)	0.6361 (3)	0.1119 (14)	0.929 (6)
F3''	0.637 (6)	0.850 (3)	0.573 (3)	0.096 (12)	0.071 (6)
C13	0.4653 (5)	0.7924 (3)	0.8855 (3)	0.0643 (9)	
O13	0.4473 (4)	0.7366 (2)	0.97198 (18)	0.0839 (8)	
C14	0.4909 (6)	0.9153 (3)	0.8704 (3)	0.0849 (12)	
H14A	0.4708	0.9407	0.9335	0.127*	
H14B	0.4046	0.9625	0.8290	0.127*	
H14C	0.6140	0.9221	0.8388	0.127*	
N1'	0.9141 (4)	0.4419 (2)	0.7688 (2)	0.0561 (7)	
N2'	0.9250 (4)	0.3460 (2)	0.8425 (2)	0.0612 (7)	
C3'	0.9172 (4)	0.2592 (3)	0.7996 (3)	0.0587 (9)	
C4'	0.9010 (4)	0.2929 (3)	0.6972 (2)	0.0578 (9)	
C5'	0.8996 (4)	0.4117 (3)	0.6816 (2)	0.0569 (8)	
O5'	0.8858 (3)	0.4856 (2)	0.59906 (17)	0.0747 (7)	
H5'	0.8542	0.4376	0.5487	0.090*	
C6'	0.9283 (4)	0.5517 (3)	0.7911 (2)	0.0536 (8)	
C7'	0.9738 (5)	0.6410 (3)	0.7169 (3)	0.0655 (9)	
H7'	0.9917	0.6317	0.6506	0.079*	
C8'	0.9925 (5)	0.7435 (3)	0.7409 (3)	0.0737 (11)	
H8'	1.0230	0.8038	0.6908	0.088*	
C9'	0.9668 (5)	0.7580 (3)	0.8383 (3)	0.0778 (11)	
H9'	0.9813	0.8276	0.8542	0.093*	
C10'	0.9198 (5)	0.6702 (3)	0.9119 (3)	0.0768 (11)	
H10'	0.9013	0.6805	0.9779	0.092*	
C11'	0.8993 (5)	0.5662 (3)	0.8896 (3)	0.0652 (9)	
H11'	0.8666	0.5067	0.9399	0.078*	
C12'	0.9344 (6)	0.1406 (3)	0.8617 (3)	0.0736 (10)	
F1'	1.0741 (4)	0.06809 (19)	0.8220 (2)	0.1054 (8)	
F2'	0.9629 (4)	0.14090 (19)	0.95283 (18)	0.1084 (9)	
F3'	0.7860 (3)	0.09262 (18)	0.86974 (18)	0.0964 (8)	
C13'	0.8886 (5)	0.2391 (3)	0.6145 (3)	0.0726 (10)	
O13'	0.8789 (4)	0.3019 (3)	0.5317 (2)	0.0953 (9)	
C14'	0.8891 (7)	0.1121 (3)	0.6241 (3)	0.0989 (14)	
H14D	0.8621	0.0961	0.5635	0.148*	
H14E	0.7975	0.0886	0.6780	0.148*	
H14F	1.0081	0.0701	0.6371	0.148*	

### Atomic displacement parameters (Å^2^)

**Table:**

	*U*^11^	*U*^22^	*U*^33^	*U*^12^	*U*^13^	*U*^23^
N1	0.0614 (17)	0.0456 (15)	0.0463 (16)	−0.0126 (12)	−0.0084 (13)	0.0007 (12)
N2	0.077 (2)	0.0473 (16)	0.0473 (16)	−0.0152 (13)	−0.0068 (13)	0.0045 (13)
C3	0.068 (2)	0.0430 (18)	0.054 (2)	−0.0103 (16)	−0.0102 (17)	−0.0010 (16)
C4	0.055 (2)	0.0486 (18)	0.053 (2)	−0.0092 (15)	−0.0099 (15)	−0.0037 (15)
C5	0.055 (2)	0.056 (2)	0.0487 (19)	−0.0117 (16)	−0.0067 (15)	−0.0035 (16)
O5	0.0961 (19)	0.0660 (15)	0.0460 (13)	−0.0251 (13)	−0.0112 (12)	0.0044 (11)
C6	0.050 (2)	0.0445 (18)	0.056 (2)	−0.0085 (14)	−0.0029 (15)	−0.0060 (15)
C7	0.077 (3)	0.055 (2)	0.066 (2)	−0.0159 (18)	−0.0112 (19)	0.0008 (18)
C8	0.084 (3)	0.050 (2)	0.086 (3)	−0.0157 (19)	−0.011 (2)	0.002 (2)
C9	0.073 (3)	0.052 (2)	0.098 (3)	−0.0164 (18)	−0.002 (2)	−0.019 (2)
C10	0.086 (3)	0.064 (2)	0.070 (2)	−0.022 (2)	−0.006 (2)	−0.018 (2)
C11	0.079 (3)	0.054 (2)	0.057 (2)	−0.0170 (18)	−0.0042 (18)	−0.0073 (17)
C12	0.107 (4)	0.053 (2)	0.064 (3)	−0.017 (2)	−0.013 (3)	−0.002 (2)
F1	0.244 (5)	0.0707 (18)	0.0569 (19)	−0.053 (2)	−0.025 (2)	0.0120 (14)
F1''	0.18 (2)	0.10 (2)	0.07 (2)	−0.04 (2)	−0.05 (2)	0.033 (19)
F2	0.137 (3)	0.0565 (17)	0.121 (3)	0.0069 (18)	−0.022 (2)	0.0097 (16)
F2''	0.15 (3)	0.059 (18)	0.12 (2)	−0.049 (19)	−0.05 (2)	0.007 (16)
F3	0.118 (3)	0.087 (2)	0.135 (3)	−0.059 (2)	−0.030 (2)	0.023 (2)
F3''	0.14 (2)	0.060 (18)	0.08 (2)	−0.040 (17)	−0.042 (19)	0.052 (16)
C13	0.064 (2)	0.062 (2)	0.069 (2)	−0.0067 (17)	−0.0114 (18)	−0.0165 (19)
O13	0.118 (2)	0.0789 (18)	0.0542 (16)	−0.0138 (15)	−0.0083 (15)	−0.0153 (14)
C14	0.107 (3)	0.064 (2)	0.093 (3)	−0.014 (2)	−0.018 (2)	−0.032 (2)
N1'	0.0623 (18)	0.0467 (16)	0.0565 (17)	−0.0101 (13)	−0.0075 (14)	−0.0023 (13)
N2'	0.0710 (19)	0.0489 (17)	0.0611 (18)	−0.0104 (14)	−0.0097 (14)	−0.0021 (14)
C3'	0.057 (2)	0.049 (2)	0.070 (2)	−0.0112 (16)	−0.0084 (17)	−0.0059 (17)
C4'	0.057 (2)	0.057 (2)	0.061 (2)	−0.0148 (16)	−0.0053 (17)	−0.0095 (17)
C5'	0.053 (2)	0.061 (2)	0.054 (2)	−0.0096 (16)	−0.0055 (16)	−0.0046 (17)
O5'	0.0982 (19)	0.0699 (16)	0.0563 (15)	−0.0246 (14)	−0.0168 (13)	0.0045 (13)
C6'	0.0479 (19)	0.0483 (19)	0.062 (2)	−0.0051 (15)	−0.0083 (16)	−0.0067 (16)
C7'	0.070 (2)	0.054 (2)	0.067 (2)	−0.0113 (17)	−0.0032 (18)	−0.0036 (18)
C8'	0.077 (3)	0.050 (2)	0.088 (3)	−0.0139 (18)	−0.008 (2)	0.002 (2)
C9'	0.080 (3)	0.057 (2)	0.102 (3)	−0.0088 (19)	−0.022 (2)	−0.020 (2)
C10'	0.089 (3)	0.065 (3)	0.080 (3)	−0.008 (2)	−0.021 (2)	−0.019 (2)
C11'	0.072 (2)	0.058 (2)	0.064 (2)	−0.0108 (18)	−0.0118 (18)	−0.0035 (18)
C12'	0.086 (3)	0.056 (2)	0.080 (3)	−0.015 (2)	−0.017 (2)	−0.008 (2)
F1'	0.1063 (19)	0.0639 (14)	0.130 (2)	0.0090 (13)	−0.0078 (16)	−0.0090 (14)
F2'	0.175 (3)	0.0663 (14)	0.0878 (18)	−0.0275 (15)	−0.0512 (17)	0.0124 (13)
F3'	0.1083 (19)	0.0687 (14)	0.1132 (18)	−0.0376 (13)	−0.0114 (14)	0.0012 (13)
C13'	0.073 (3)	0.075 (3)	0.075 (3)	−0.024 (2)	−0.006 (2)	−0.016 (2)
O13'	0.131 (3)	0.098 (2)	0.0654 (18)	−0.0395 (18)	−0.0116 (17)	−0.0173 (16)
C14'	0.125 (4)	0.083 (3)	0.104 (3)	−0.031 (3)	−0.018 (3)	−0.036 (3)

### Geometric parameters (Å, °)

**Table:**

N1—C5	1.353 (4)	C14—H14C	0.9600
N1—N2	1.378 (3)	N1'—C5'	1.354 (4)
N1—C6	1.432 (4)	N1'—N2'	1.374 (3)
N2—C3	1.312 (4)	N1'—C6'	1.432 (4)
C3—C4	1.413 (4)	N2'—C3'	1.308 (4)
C3—C12	1.488 (5)	C3'—C4'	1.416 (5)
C4—C5	1.392 (4)	C3'—C12'	1.499 (5)
C4—C13	1.440 (5)	C4'—C5'	1.390 (4)
C5—O5	1.311 (4)	C4'—C13'	1.445 (5)
O5—H5	1.1757	C5'—O5'	1.306 (4)
C6—C11	1.375 (4)	O5'—H5'	1.0662
C6—C7	1.376 (4)	C6'—C7'	1.377 (4)
C7—C8	1.377 (5)	C6'—C11'	1.385 (4)
C7—H7	0.9300	C7'—C8'	1.368 (5)
C8—C9	1.364 (5)	C7'—H7'	0.9300
C8—H8	0.9300	C8'—C9'	1.371 (5)
C9—C10	1.373 (5)	C8'—H8'	0.9300
C9—H9	0.9300	C9'—C10'	1.364 (5)
C10—C11	1.383 (4)	C9'—H9'	0.9300
C10—H10	0.9300	C10'—C11'	1.380 (5)
C11—H11	0.9300	C10'—H10'	0.9300
C12—F3''	1.22 (3)	C11'—H11'	0.9300
C12—F1''	1.24 (3)	C12'—F2'	1.317 (4)
C12—F2''	1.26 (3)	C12'—F3'	1.324 (4)
C12—F3	1.315 (5)	C12'—F1'	1.335 (4)
C12—F1	1.323 (5)	C13'—O13'	1.246 (4)
C12—F2	1.331 (5)	C13'—C14'	1.494 (5)
C13—O13	1.249 (4)	C14'—H14D	0.9600
C13—C14	1.486 (5)	C14'—H14E	0.9600
C14—H14A	0.9600	C14'—H14F	0.9600
C14—H14B	0.9600		
			
C5—N1—N2	109.9 (2)	C13—C14—H14C	109.5
C5—N1—C6	130.8 (3)	H14A—C14—H14C	109.5
N2—N1—C6	119.3 (2)	H14B—C14—H14C	109.5
C3—N2—N1	105.6 (2)	C5'—N1'—N2'	110.4 (3)
N2—C3—C4	112.9 (3)	C5'—N1'—C6'	130.3 (3)
N2—C3—C12	116.8 (3)	N2'—N1'—C6'	119.2 (3)
C4—C3—C12	130.3 (3)	C3'—N2'—N1'	105.4 (3)
C5—C4—C3	102.8 (3)	N2'—C3'—C4'	113.0 (3)
C5—C4—C13	119.1 (3)	N2'—C3'—C12'	117.5 (3)
C3—C4—C13	138.1 (3)	C4'—C3'—C12'	129.4 (3)
O5—C5—N1	123.7 (3)	C5'—C4'—C3'	102.8 (3)
O5—C5—C4	127.5 (3)	C5'—C4'—C13'	119.1 (3)
N1—C5—C4	108.8 (3)	C3'—C4'—C13'	138.1 (3)
C5—O5—H5	100.5	O5'—C5'—N1'	123.6 (3)
C11—C6—C7	120.5 (3)	O5'—C5'—C4'	127.9 (3)
C11—C6—N1	118.4 (3)	N1'—C5'—C4'	108.4 (3)
C7—C6—N1	121.1 (3)	C5'—O5'—H5'	104.8
C6—C7—C8	119.0 (3)	C7'—C6'—C11'	120.1 (3)
C6—C7—H7	120.5	C7'—C6'—N1'	121.2 (3)
C8—C7—H7	120.5	C11'—C6'—N1'	118.7 (3)
C9—C8—C7	121.4 (4)	C8'—C7'—C6'	119.8 (3)
C9—C8—H8	119.3	C8'—C7'—H7'	120.1
C7—C8—H8	119.3	C6'—C7'—H7'	120.1
C8—C9—C10	119.0 (3)	C7'—C8'—C9'	120.6 (3)
C8—C9—H9	120.5	C7'—C8'—H8'	119.7
C10—C9—H9	120.5	C9'—C8'—H8'	119.7
C9—C10—C11	120.8 (4)	C10'—C9'—C8'	119.8 (4)
C9—C10—H10	119.6	C10'—C9'—H9'	120.1
C11—C10—H10	119.6	C8'—C9'—H9'	120.1
C6—C11—C10	119.2 (3)	C9'—C10'—C11'	120.7 (4)
C6—C11—H11	120.4	C9'—C10'—H10'	119.6
C10—C11—H11	120.4	C11'—C10'—H10'	119.6
F3''—C12—F1''	110.4 (15)	C10'—C11'—C6'	119.1 (3)
F3''—C12—F2''	106.8 (15)	C10'—C11'—H11'	120.5
F1''—C12—F2''	105.9 (17)	C6'—C11'—H11'	120.5
F3—C12—F1	107.1 (4)	F2'—C12'—F3'	106.7 (3)
F3—C12—F2	106.0 (4)	F2'—C12'—F1'	107.2 (3)
F1—C12—F2	105.5 (4)	F3'—C12'—F1'	105.9 (3)
F3''—C12—C3	107.1 (14)	F2'—C12'—C3'	112.6 (3)
F1''—C12—C3	109.2 (19)	F3'—C12'—C3'	112.7 (3)
F2''—C12—C3	117.4 (19)	F1'—C12'—C3'	111.3 (3)
F3—C12—C3	113.5 (4)	O13'—C13'—C4'	118.0 (3)
F1—C12—C3	113.1 (3)	O13'—C13'—C14'	119.2 (4)
F2—C12—C3	111.1 (3)	C4'—C13'—C14'	122.9 (4)
O13—C13—C4	117.9 (3)	C13'—C14'—H14D	109.5
O13—C13—C14	118.9 (3)	C13'—C14'—H14E	109.5
C4—C13—C14	123.1 (3)	H14D—C14'—H14E	109.5
C13—C14—H14A	109.5	C13'—C14'—H14F	109.5
C13—C14—H14B	109.5	H14D—C14'—H14F	109.5
H14A—C14—H14B	109.5	H14E—C14'—H14F	109.5
			
C5—N1—N2—C3	0.0 (3)	C3—C4—C13—O13	179.8 (4)
C6—N1—N2—C3	178.1 (3)	C5—C4—C13—C14	179.1 (3)
N1—N2—C3—C4	0.4 (4)	C3—C4—C13—C14	−0.8 (6)
N1—N2—C3—C12	−178.7 (3)	C5'—N1'—N2'—C3'	0.1 (3)
N2—C3—C4—C5	−0.6 (4)	C6'—N1'—N2'—C3'	−176.9 (3)
C12—C3—C4—C5	178.3 (4)	N1'—N2'—C3'—C4'	0.0 (4)
N2—C3—C4—C13	179.3 (4)	N1'—N2'—C3'—C12'	177.4 (3)
C12—C3—C4—C13	−1.9 (7)	N2'—C3'—C4'—C5'	0.0 (4)
N2—N1—C5—O5	178.9 (3)	C12'—C3'—C4'—C5'	−177.1 (3)
C6—N1—C5—O5	1.1 (5)	N2'—C3'—C4'—C13'	179.4 (4)
N2—N1—C5—C4	−0.4 (4)	C12'—C3'—C4'—C13'	2.3 (7)
C6—N1—C5—C4	−178.2 (3)	N2'—N1'—C5'—O5'	179.5 (3)
C3—C4—C5—O5	−178.6 (3)	C6'—N1'—C5'—O5'	−3.9 (5)
C13—C4—C5—O5	1.4 (5)	N2'—N1'—C5'—C4'	−0.1 (4)
C3—C4—C5—N1	0.6 (3)	C6'—N1'—C5'—C4'	176.5 (3)
C13—C4—C5—N1	−179.3 (3)	C3'—C4'—C5'—O5'	−179.5 (3)
C5—N1—C6—C11	167.1 (3)	C13'—C4'—C5'—O5'	0.9 (5)
N2—N1—C6—C11	−10.6 (4)	C3'—C4'—C5'—N1'	0.1 (4)
C5—N1—C6—C7	−13.6 (5)	C13'—C4'—C5'—N1'	−179.5 (3)
N2—N1—C6—C7	168.8 (3)	C5'—N1'—C6'—C7'	−16.1 (5)
C11—C6—C7—C8	−0.2 (5)	N2'—N1'—C6'—C7'	160.2 (3)
N1—C6—C7—C8	−179.6 (3)	C5'—N1'—C6'—C11'	165.3 (3)
C6—C7—C8—C9	0.9 (6)	N2'—N1'—C6'—C11'	−18.4 (4)
C7—C8—C9—C10	−1.0 (6)	C11'—C6'—C7'—C8'	0.8 (5)
C8—C9—C10—C11	0.5 (6)	N1'—C6'—C7'—C8'	−177.7 (3)
C7—C6—C11—C10	−0.3 (5)	C6'—C7'—C8'—C9'	0.1 (6)
N1—C6—C11—C10	179.0 (3)	C7'—C8'—C9'—C10'	−0.8 (6)
C9—C10—C11—C6	0.2 (5)	C8'—C9'—C10'—C11'	0.6 (6)
N2—C3—C12—F3''	−59 (3)	C9'—C10'—C11'—C6'	0.4 (6)
C4—C3—C12—F3''	122 (3)	C7'—C6'—C11'—C10'	−1.1 (5)
N2—C3—C12—F1''	60 (3)	N1'—C6'—C11'—C10'	177.5 (3)
C4—C3—C12—F1''	−119 (3)	N2'—C3'—C12'—F2'	−2.5 (5)
N2—C3—C12—F2''	−179 (3)	C4'—C3'—C12'—F2'	174.5 (4)
C4—C3—C12—F2''	2(3)	N2'—C3'—C12'—F3'	118.3 (4)
N2—C3—C12—F3	−122.2 (5)	C4'—C3'—C12'—F3'	−64.8 (5)
C4—C3—C12—F3	59.0 (6)	N2'—C3'—C12'—F1'	−122.9 (4)
N2—C3—C12—F1	0.0 (6)	C4'—C3'—C12'—F1'	54.0 (5)
C4—C3—C12—F1	−178.9 (4)	C5'—C4'—C13'—O13'	0.7 (5)
N2—C3—C12—F2	118.4 (4)	C3'—C4'—C13'—O13'	−178.6 (4)
C4—C3—C12—F2	−60.4 (6)	C5'—C4'—C13'—C14'	180.0 (4)
C5—C4—C13—O13	−0.3 (5)	C3'—C4'—C13'—C14'	0.7 (7)

### Hydrogen-bond geometry (Å, °)

**Table:**

*D*—H···*A*	*D*—H	H···*A*	*D*···*A*	*D*—H···*A*
O5—H5···O13	1.18	1.60	2.548 (3)	132
O5'—H5'···O13'	1.07	1.65	2.560 (4)	139
C14—H14B···F1'^i^	0.96	2.55	3.394 (5)	146
C7—H7···O13^ii^	0.93	2.64	3.446 (4)	145
C7'—H7'···O13'^iii^	0.93	2.57	3.406 (4)	149

Symmetry codes: (i) *x*−1, *y*+1, *z*; (ii) −*x*+1, −*y*+1, −*z*+2; (iii) −*x*+2, −*y*+1, −*z*+1.
